# Recycling of a Biodegradable Polymer Blend

**DOI:** 10.3390/polym12102297

**Published:** 2020-10-08

**Authors:** Francesco Paolo La Mantia, Luigi Botta, Maria Chiara Mistretta, Antonino Di Fiore, Vincenzo Titone

**Affiliations:** 1 Department of Engineering, University of Palermo, Viale delle Scienze, 90128 Palermo, Italy; luigi.botta@unipa.it (L.B.); mariachiara.mistretta@unipa.it (M.C.M.); ninodifiore@hotmail.it (A.D.F.); vincenzo.titone@unipa.it (V.T.); 2 INSTM, Via Giusti 9, 50125 Firenze, Italy; 3 Joeplast, 92025 Casteltermini, Italy

**Keywords:** bioplastic, extrusion, mechanical recycling, poly(butylene adipate-co-terephthalate), poly(lactic acid)

## Abstract

Mechanical recycling is one of the possible ways to enhance the value of postconsumer plastic materials. However, the final performance of the recycled material will strongly depend on the quality of the selection made on the recycled product and on the degradation of the properties. In this context, the present study examines the effect of reprocessing for five successive extrusion cycles on the rheological, mechanical and thermal properties of a poly(butylene adipate-co-terephthalate) (PBAT)-based blend on samples reprocessed in both dry and wet conditions. The results showed that when the sample was processed after drying, degradation of the material was less than when it was processed in wet conditions. However, the experimental results showed that the decrease of rheological and mechanical properties was not so dramatic, and therefore the material could be reused in both cases.

## 1. Introduction

Although the so-called bioplastics, i.e., biodegradable polymers, represent only a very small amount of the plastic materials produced in the world [1] and although the obvious final fate of these polymers is biodegradation with formation of carbon dioxide, water and biomass [2,3,4,5,6], mechanical recycling could be an interesting intermediate step. Indeed, mechanical recycling could be an interesting tool for exploiting the same polymers more often if the properties of the recycled biodegradable polymers are still suitable for some applications.

In previous reviews [7,8,9], the main degradation pathways of several bioplastics, including the most important bioplastics, were reviewed. It was shown that mechanical recycling provides several advantages over chemical recycling, particularly low cost and, especially, a reduced environmental impact. Nevertheless, only a few works discuss the mechanical recycling of biodegradable polymers. 

La Mantia et al. [10] investigated the rheological and mechanical properties of a biodegradable, starch-based polymer after multiple processing steps in an extruder. The results showed that the adopted reprocessing conditions did not significantly influence the rheological and mechanical properties of the sample; thus, it was found that the material could be reprocessed with the same conditions used for the untreated sample. Similarly, Morreale et al. [11] observed no significant loss of mechanical properties in green composites from biopolymer matrix (Bioflex) and wood flour as a result of multiple processing cycles. Beltran et al. [12,13] studied the reprocessing effect on the properties of postconsumer poly(lactic acid) (PLA) subjected to multiple extrusion cycles. The results showed that recycled PLA with good properties can be obtained by adding small amounts of additives during mechanical recycling. Different approaches concern the possible use of additives that restore the molecular weight of the polymer phase. Barletta et al. [14] studied the use of a chain extender in the PLA recycling of PLA-based bioplastics. This study showed that the repeated extrusion cycles caused a decrease of the polymers’ molecular weight. However, smaller variations of the melt flow index (MFI) values with recycling were recorded in cases where a chain extender was added to the compounds, since the reaction of functional groups of the additive with the polymer terminal molecules promoted branching between polymeric chains, partially restoring the molecular weight. 

Of course, the degradation undergone during processing and the environmental degradation during the lifetime are the main hindrances to the recycling of these polymers. It is, indeed, well known that these biodegradable polymers are quite labile with respect to both thermomechanical degradation [15,16,17,18] and photo-oxidation [19,20,21,22,23,24]. 

The aim of this work was to evaluate the effect of reprocessing in a single-screw extruder on the rheological and mechanical properties of a commercial, biodegradable blend based on poly(butylene adipate-co-terephthalate) (PBAT) and poly(lactic acid) (PLA). In particular, rheological and mechanical tests were carried out on materials reprocessed by means of five subsequent extrusion cycles. Moreover, extrusion cycles were carried out on both dry and wet samples. 

## 2. Materials and Methods

### 2.1. Material and Reprocessing

The biodegradable polymer blend used in this work is a commercial PLA/PBAT blend supplied by BASF (Ludwigshafen, Germany) under the commercial name Ecovio, grade F23B1. In this commercial grade, PBAT is the predominant phase with about 12% calcium carbonate [25]. 

The reprocessing of Ecovio was carried out using a single screw extruder (Thermo Scientific HAAKE PolyLab QC, Karlsruhe, Germany) for five subsequent extrusion cycles. The temperature profile was set to 150–160–170–180 °C, while the rotational speed of the screw was 30 rpm. Extrusion tests were carried out on both dry and wet samples. In the first case—dry—before each extrusion, the material was dried overnight under vacuum at 70 °C. In the second case—wet—the samples were kept in environmental conditions (about 23–25 °C and 60–70% RH) for about 12 h. The moisture content was 0.15%.

The sample codes of all the samples are reported in Table 1 together with their treatment.

Part of the molten polymer coming out from the extruder die was cooled in air and afterwards pelletized to be used for further extrusions and characterizations. 

### 2.2. Rheological, Mechanical and Structural Characterization

The rheological characterization was performed at 180 °C using both an ARES G2 (TA Instruments, New Castle, DE, USA) plate–plate rotational rheometer and a capillary viscometer. The specimens for the measurements with the rheometer were obtained by compression molding. CEAST’s Rheologic 1000 capillary viscometer (CEAST, Torino, Italy) was used for the viscosity measurements in the higher range of shear rates. As this polymer is used in film blowing, the rheological behavior in non-isothermal, elongational flow was also tested using the same capillary viscometer equipped with a drawing system. The force at break of the molten filament was read directly and is known as melt strength (MS). The breaking stretching ratio (BSR) was calculated as the ratio between the drawing speed at break and the extrusion speed at the die [26,27,28]. The specimens were carefully dried before testing.

Tensile measurements were carried out using an Instron Model 3365 universal testing machine (according to ASTM D638), (Instron, High Wycombe, UK) on rectangular specimens (10 × 90 × 0.4 mm^3^) cut off from sheets prepared by compression molding at 180 °C under a mold pressure of 5000 psi; holding time, 3 min; cooling time, 10 min. The specimens for the tensile tests were equilibrated to the environmental humidity for at least 12 h.

Thermal properties of the materials were evaluated by differential scanning calorimetry (DSC). DSC measurements were performed by a DSC-60 Shimadzu (Shimadzu, Kyoto, Japan), under nitrogen gas atmosphere, using 10 ± 2 mg of sample and 10 °C/min heating rate up to 200 °C/min. 

Degree of cross-linking was determined using a Soxhlet extractor and using a tetrahydrofuran (THF)-like solvent for 48 h. 

## 3. Results and Discussion

**Figure 1:**
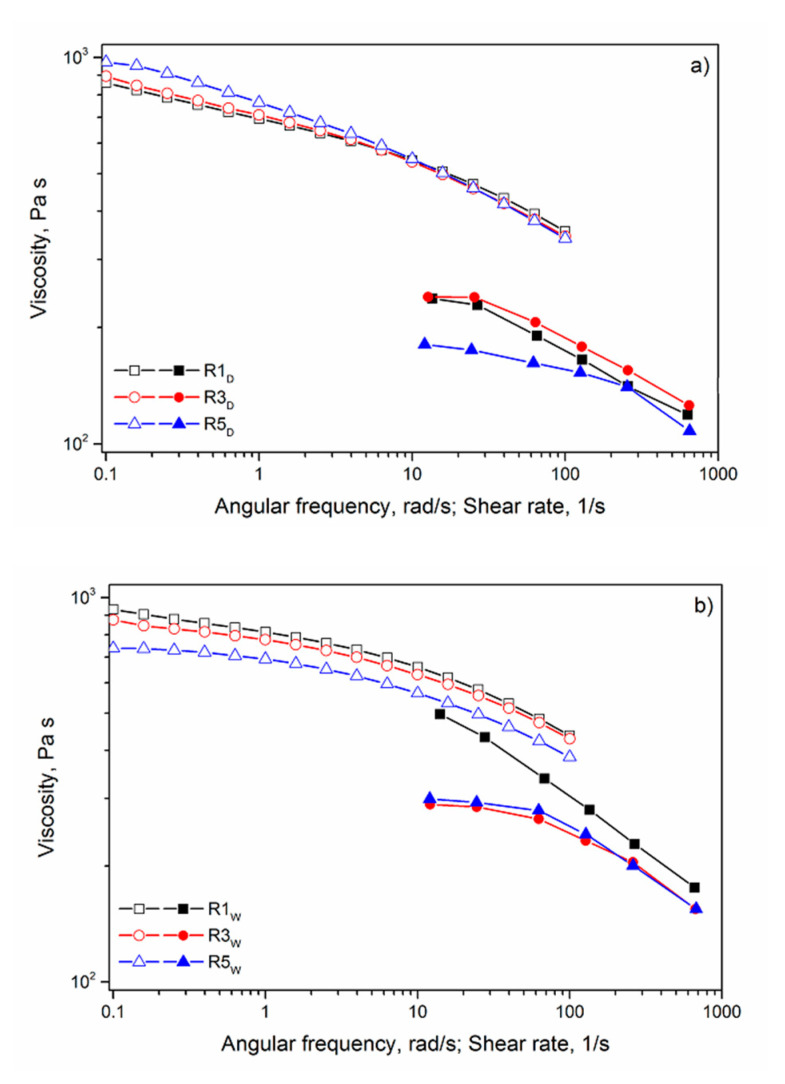
Flow curves of the Ecovio after 1, 3, and 5 extrusions steps for (**a**) dried sample and (**b**) wet sample. Data taken from rotational rheometer (open symbols) and capillary viscometer (closed symbols).

In Figure 1, the flow curves of the Ecovio, virgin and reprocessed three and five times, are reported for the samples processed in dry and wet conditions. The first feature to be observed was that the flow curves obtained in the rheometer and the flow curves measured in the capillary viscometer do not superimpose as predicted by the Cox–Merz rule. This means that the Cox–Merz equation did not hold for these systems. This feature was already found for the same Mater-Bi sample [29] and for other systems [30,31,32] and explained considering the multiphase nature of these polymer systems.

When the sample was processed after the drying step, the viscosity slightly increased, particularly at low frequencies with increasing number of extrusion steps. On the contrary, when the polymer was processed in wet conditions, the viscosity of the sample decreased with increasing number of extrusions. 

**Figure 2:**
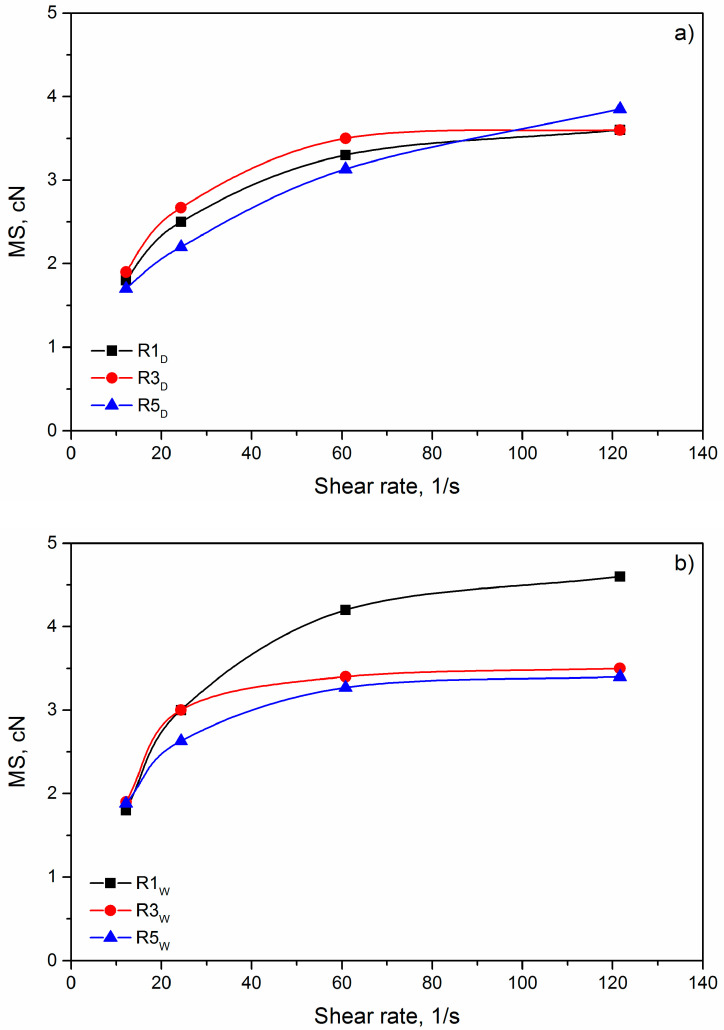
Melt strength (MS) as a function of the apparent shear rate for (**a**) dried sample and (**b**) wet sample.

The melt strength (MS), i.e., the force at break in the molten filament, is reported in Figure 2 as a function of the apparent shear rate in the capillary viscometer. MS behavior is similar to that found for the viscosity. Indeed, when the test was carried out on the sample processed in dry conditions, MS increased slightly with the number of reprocessing steps. As for the melt strength values for the sample processed in wet conditions, the MS shear rate curves decreased for the reprocessed samples. MS behavior for the dry samples was slightly different than that observed for the flow curves in the low frequency range. This was due to the fact that the tests in non-isothermal elongational flow were carried out in the relatively high shear rate range when the viscosity of all three samples was about the same (see Figure 1a). The melt strength was strictly related to the melt viscosity, and this explained the behavior of the MS curves for the wet sample. 

**Figure 3:**
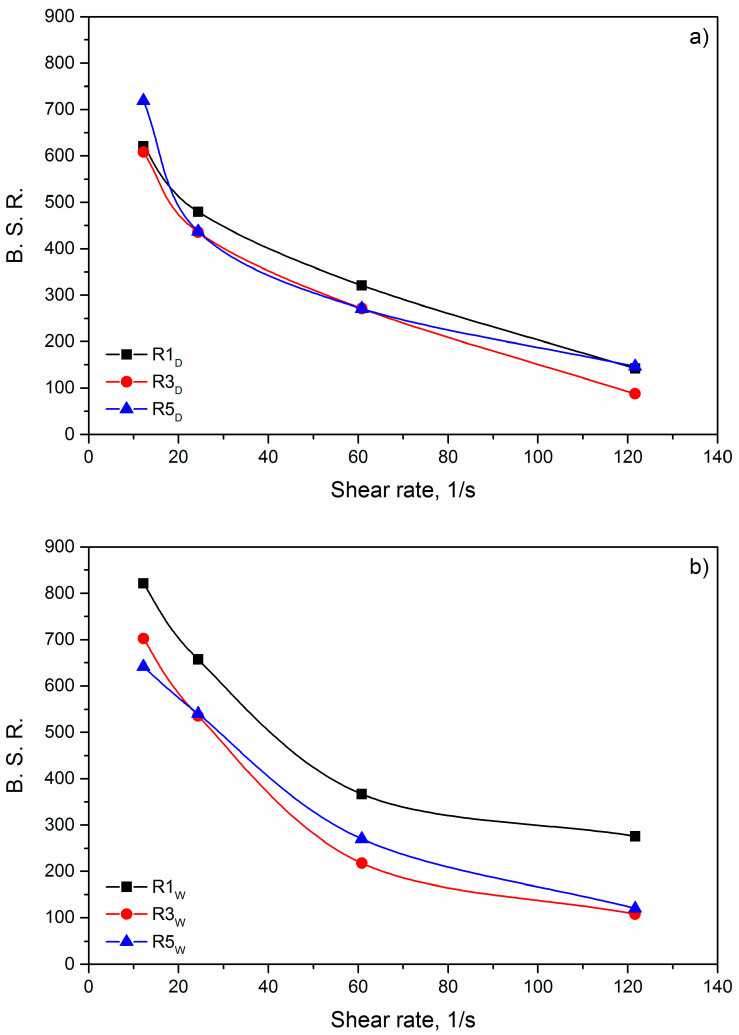
Breaking stretching ratio (BSR) as a function of the apparent shear rate for (**a**) dried sample and (**b**) wet sample.

The breaking stretching ratio (BSR) as a function of the apparent shear rate is reported in Figure 3. The BSR curves present a mirror image of the MS curves as they decrease with increasing shear rate.

The deformability of the molten filament slightly decreased for the sample reprocessed after drying, and the samples reprocessed three and five times showed about the same BSR curves. Similar behavior was shown in the BSR curves of the sample reprocessed in wet conditions, but the decrease of the BSR values was larger.

The decrease of the viscosity is, of course, correlated with a small reduction of the molecular weight because of the thermomechanical degradation during the reprocessing steps. The increase of the viscosity must be, then, correlated with an increase of the molecular weight or with a formation of some cross-links or some branching during the reprocessing. PBAT is, indeed, prone to form crosslinks during degradation [19,20,21,33], and the branching is the first step in forming cross-linked structures. No cross-links were revealed for the PLA. In the reprocessed samples, no cross-linking was found, so the increase of viscosity can be correlated only with the presence of branched macromolecules formed because of the thermomechanical stress. 

**Scheme 1:**
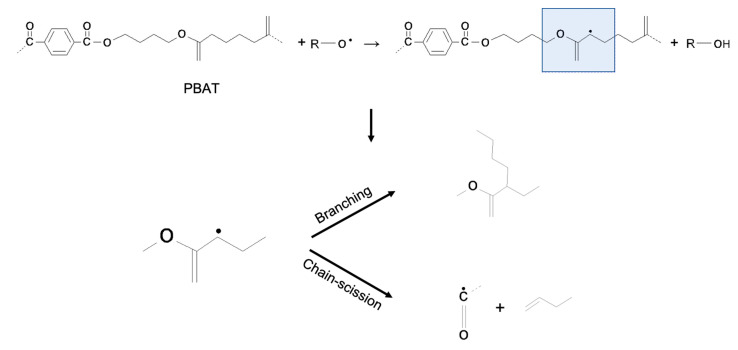
Proposed reaction mechanics.

A possible scheme of the two mechanisms of degradation of PBAT is reported in Scheme 1. The carbon of the methyl group near the C=O group is prone to be attacked by a free radical, giving rise to a radical that can lead to both branching and chain scission. 

Of course, both chain scission and branching occurred during the reprocessing cycle, but for the sample reprocessed in wet conditions, the chain scission was predominant. For the dried sample, it seems that the branching was predominant. 

**Figure 4:**
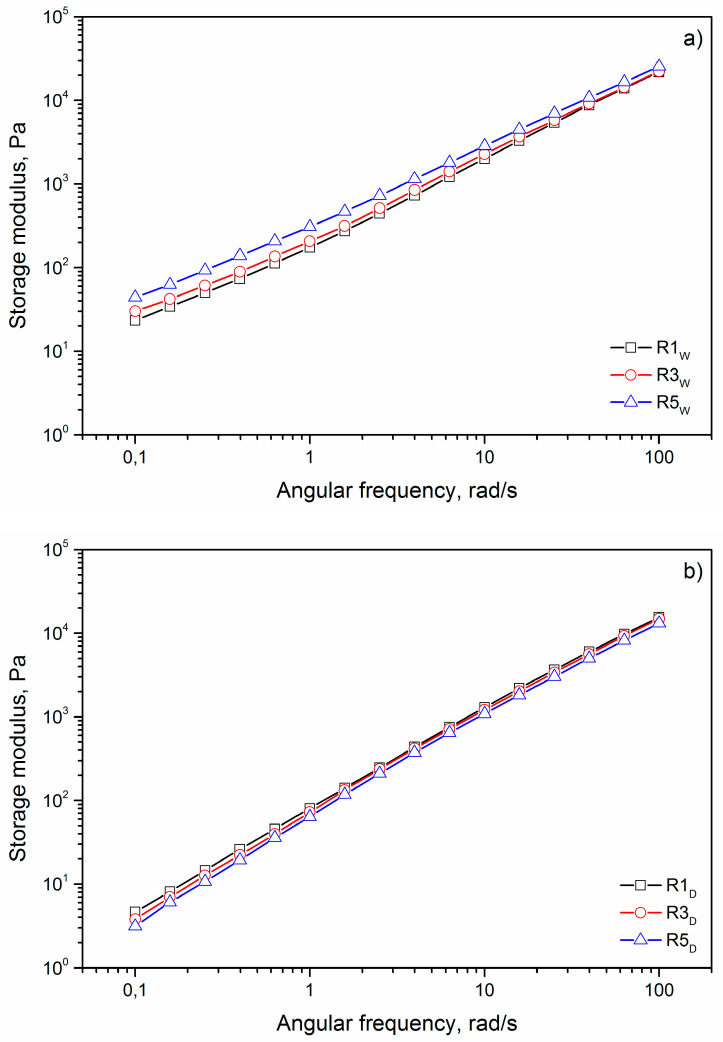
Storage modulus as a function of the frequency for (**a**) dry sample and (**b**) wet sample.

This interpretation is confirmed by the storage modulus curves reported in Figure 4a for the sample reprocessed after drying and in Figure 4b for the sample reprocessed without drying. The decrease of the slope in the low frequency range is correlated with a more "solid-like" behavior of the sample because, for a solid-elastic material, the slope should be zero.

On the contrary, no significant difference in the slope was observed for the G’ curve of the sample reprocessed in wet conditions. 

In a previous work [33], it was demonstrated that photoxidation of PBAT can lead to different levels of cross-linking, depending on the environmental conditions. In particular, when photooxidation was carried out with a condensation cycle, cross-linking was lower than that measured when photooxidation was carried out without a condensation cycle. This behavior was interpreted in terms of a significant contribution to the reduction of molecular weight of the hydrolysis of the copolyesters. The two processes, reduction of molecular weight and possible branching, are in competition, but the hydrolysis due to the presence of water shifted this competition towards the reduction of molecular weight, while the opposite was true when the reprocessing was carried out on predried samples.

The mechanical properties, namely elastic modulus (E), tensile strength (TS) and elongation at break (EB), of unprocessed Ecovio are reported in Table 2 [20].

**Figure 5:**
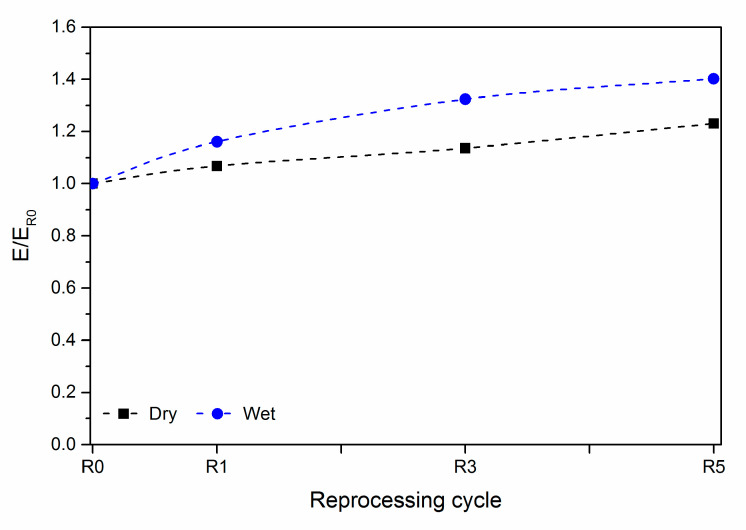
Dimensionless elastic modulus as a function of the reprocessing cycles.

**Figure 6:**
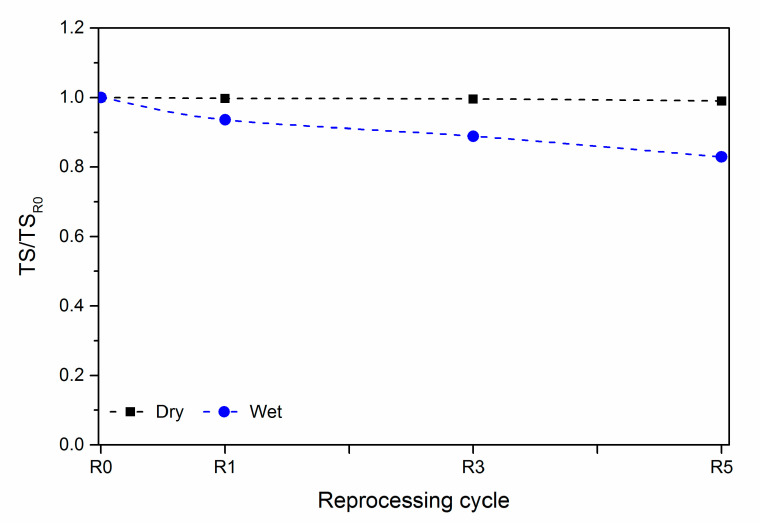
Dimensionless tensile strength as a function of the reprocessing cycles.

**Figure 7:**
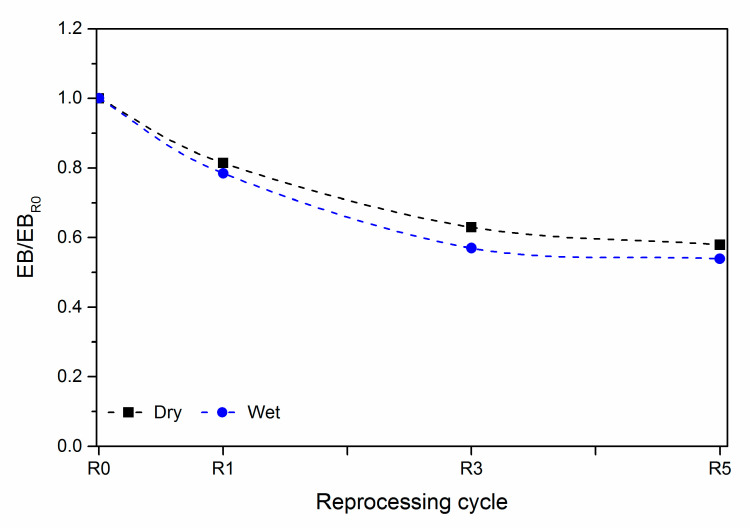
Dimensionless elongation at break as a function of the reprocessing cycles.

In Figure 5, Figure 6 and Figure 7, the dimensionless values of the same mechanical properties are reported as a function of the reprocessing steps. The dimensionless values were obtained by dividing the values at a given recycling cycle by the value of the virgin polymer. For both reprocessing conditions, the elastic modulus increased while the elongation at break decreased with the number of extrusions. The tensile strength was almost constant or showed a slight decrease. While the reduction of the elongation at break can be ascribed to the reduction of the molecular weight during the reprocessing steps, the increase of the modulus was intepreted in previoius works [34,35] as the result of the increase of crystallinity due to the decrease of the molecular weight.

The increase of the modulus and then of the rigidity should lead to some increase of the tensile strength; however, the tensile strength was observed to decrease slightly. This behavior can be interpreted in terms of premature rupture of the specimen because of the reduction of the elongation at break.

**Figure 8:**
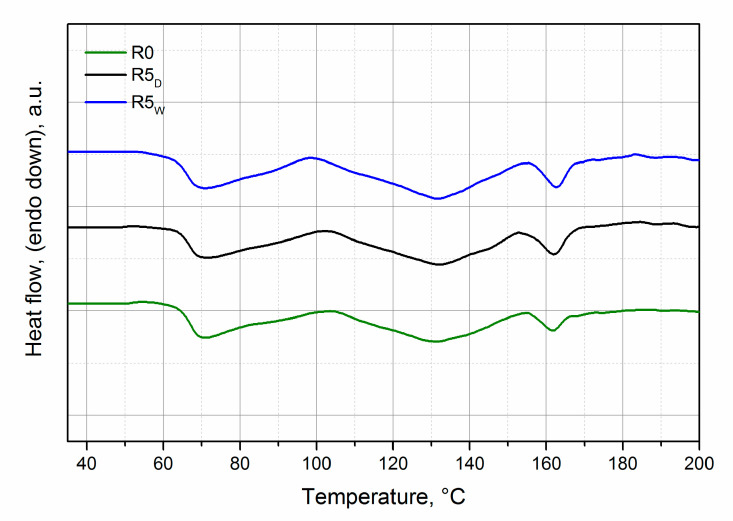
Thermograms of the virgin sample and of the samples reprocessed 5 times in dry and wet conditions.

The increase of the crystallinity, responsible for the increase of the elastic modulus, was confirmed by thermograms of the virgin sample and of the samples reprocessed five times in dry and wet conditions (reported in Figure 8). The thermograms refer to the first heating in order to take into account the morphological variations that occurred in the sample due to the thermomechanical process. Although it is not possible to draw from the thermograms the values of the crystallinity of the two components because the formulation and the composition of the blend is not known, it is evident that the reprocessing cycles slightly increased both peaks of fusion of the two polymers (Table 3). The peak at lower temperature refers to the PBAT and the peak at higher temperature to the PLA. For the PBAT, the larger increase of crystallinity (Table 3) occurred when the reprocessing was carried out without any predrying. This was the result of the larger decrease of the molecular weight due to thermomechanical degradation and hydrolysis. As for the PLA, the enthalpy of fusion was very small, and the difference between the two samples was very small and within the experimental error. In conclusion, the increase of crystallinity occured mainly in the PBAT phase and, in particular, in wet conditions. In agreement with the previous experimeantal results, this means that the decrease of the molecular weight of the PBAT seemed more remarkable than that of the PLA, and a more marked decrease occured when the sample was not dried. 

## 4. Conclusions

In this work, the effect of the reprocessing on the rheological, mechanical and thermal properties of a biodegradable PBAT-based blend was studied. The polymer was reprocessed both after predrying and without predrying. The rheological characterization showed that when the sample was processed after drying, a lower degradation was observed because the presence of water also induced the hydrolysis of the components, in addition to the thermomechanical degradation. In particular, in the samples reprocessed after drying, branching in the PBAT majority component became slightly predominant with respect to the chain rupture, while the opposite was observed for the samples reprocessed in wet conditions.

Moreover, in both conditions, five extrusion steps did not significantly decrease the mechanical properties of the blend, thus demonstrating that this biodegradable blend can, actually, be subjected to more cycles of reprocessing. Thus, all the experimental results put in evidence positive indications regarding the reprocessing of this blend.

## Figures and Tables

**Table 1:** Sample codes and treatments.

Sample Code	Treatment
R_0_	Untreated sample
R_nD_	Sample extruded n times after drying
R_nW_	Sample extruded n times without drying

**Table 2:** Mechanical properties of the virgin sample.

	E, MPa	TS, MPa	EB, %
R0	111 ± 7.8	9.3 ± 0.5	327 ± 20

**Table 3:** Melting temperature and enthalpy of fusion of the virgin sample and of the samples reprocessed 5 times in dry and wet conditions.

	T_m_ (PBAT), °C	T_m_ (PLA), °C	ΔH_m_ (PBAT), J/g	ΔH_m_ (PLA), J/g
R0	131.3	161.3	5.91 ± 0.3	0.66 ± 0.05
R5_D_	133.4	161.4	6.33 ± 0.6	1.08 ± 0.08
R5_W_	132.5	162.4	7.85 ± 0.5	1.12 ± 0.1
